# Environmental Surveillance Reveals Complex Enterovirus Circulation Patterns in Human Populations

**DOI:** 10.1093/ofid/ofy250

**Published:** 2018-10-01

**Authors:** Manasi Majumdar, Salmaan Sharif, Dimitra Klapsa, Thomas Wilton, Muhammad Masroor Alam, Maria Dolores Fernandez-Garcia, Lubna Rehman, Ghulam Mujtaba, Gina McAllister, Heli Harvala, Kate Templeton, Edward T Mee, Humayun Asghar, Kader Ndiaye, Philip D Minor, Javier Martin

**Affiliations:** 1 Division of Virology, National Institute for Biological Standards and Control (NIBSC), South Mimms, Potters Bar, Herts, United Kingdom; 2 NIH, Islamabad, Pakistan; 3 Institut Pasteur, Dakar, Senegal; 4 Edinburgh Royal Infirmary, United Kingdom; 5 World Health Organization Eastern Mediterranean Regional Office, Amman, Jordan

**Keywords:** direct detection, enterovirus pathogenesis, environmental surveillance, human enterovirus, next-generation sequencing

## Abstract

**Background:**

Enteroviruses are common human pathogens occasionally associated with severe disease, notoriously paralytic poliomyelitis caused by poliovirus. Other enterovirus serotypes such as enterovirus A71 and D68 have been linked to severe neurological syndromes. New enterovirus serotypes continue to emerge, some believed to be derived from nonhuman primates. However, little is known about the circulation patterns of many enterovirus serotypes and, in particular, the detailed enterovirus composition of sewage samples.

**Methods:**

We used a next-generation sequencing approach analyzing reverse transcriptase polymerase chain reaction products synthesized directly from sewage concentrates.

**Results:**

We determined whole-capsid genome sequences of multiple enterovirus strains from all 4 A to D species present in environmental samples from the United Kingdom, Senegal, and Pakistan.

**Conclusions:**

Our results indicate complex enterovirus circulation patterns in human populations with differences in serotype composition between samples and evidence of sustained and widespread circulation of many enterovirus serotypes. Our analyses revealed known and divergent enterovirus strains, some of public health relevance and genetically linked to clinical isolates. Enteroviruses identified in sewage included vaccine-derived poliovirus and enterovirus D-68 stains, new enterovirus A71 and coxsackievirus A16 genogroups indigenous to Pakistan, and many strains from rarely reported serotypes. We show how this approach can be used for the early detection of emerging pathogens and to improve our understanding of enterovirus circulation in humans.

Human enteroviruses (EVs) belong to the family *Picornaviridae* and are classified into 4 viral species (EV-A to EV-D) based on their genetic identity. One hundred ten different EV serotypes have been described so far [1]. The virus particles are nonenveloped and contain a single-stranded positive-sense RNA genome ~7500 nucleotides in length in which 5′ and 3′ noncoding regions flank a single open reading frame coding for structural (VP1 to VP4 capsid proteins) and nonstructural proteins (2A to 2C and 3A to 3D) [2]. EVs transmit through the fecal–oral or respiratory route and are occasionally associated with severe disease, including meningoencephalitis, flaccid paralysis, myocarditis, sepsis-like syndrome, respiratory disease, and acute hepatitis. EV serotypes such as A71 (EV-A71) and D68 (EV-D68) have recently been associated with outbreaks of neurological disease presenting high morbidity and mortality [3, 4]. EV-A71 has been responsible for large hand, foot, and mouth disease (HFMD) outbreaks associated with rare but severe cases of brainstem encephalitis mostly in the Asia-Pacific region. Other serotypes such as coxsackievirus A16 (CV-A16) have shown high prevalence in HFMD outbreaks in Asia but have only occasionally been associated with severe health problems [5]. EV-D68 has been associated with severe respiratory disease, occasionally leading to acute flaccid paralysis, and outbreaks in North America, Europe, and Asia in 2014–2016 resulted in >2000 cases, a number of severe complications, and some deaths [6, 7]. EV-A71 and EV-D68 cases have been reported in several European countries in recent years, with increasing reports of these viruses causing severe infections [8]. It is therefore essential to develop and maintain sensitive surveillance systems to detect and identify circulating EVs that are likely to be associated with neurological disease.

In addition to clinical diagnosis [9], several countries conduct environmental surveillance (ES) for poliovirus (PV), which has proven to be a very sensitive method to detect PV circulation and has helped to trace the elimination of wild and vaccine PV in some areas [10]. As nonpolio EV infections are not nationally notifiable and are rarely treated or tested, the number of EV detections is likely a considerable underestimate of the true burden of disease. Therefore, ES could also be a potential tool to estimate circulation patterns of nonpolio EVs in human populations [11–16]. However, commonly used cell culture and Sanger nucleotide sequencing methods are selective and biased and can limit the number of EV serotypes that can be identified in a sewage sample unless various cell lines are used for virus isolation or multiple polymerase chain reaction (PCR) products and/or cDNA clones are sequenced independently [12–15]. Next-generation sequencing (NGS) metagenomics and target-specific techniques have been described by us and others to detect EV strains in clinical and environmental samples [17–20]. However, the output from NGS analysis of uncultured EV strains present in sewage has been so far mostly restricted to information on the EV species or, in very few cases, the EV serotype composition [21], with insufficient nucleotide sequence information down to the genotype and strain level.

With this background in mind, we have used a novel NGS-based approach sequencing “whole-capsid region” reverse transcriptase PCR (RT-PCR) products obtained directly from sewage concentrates for the identification of multiple EVs in environmental samples. We have compared the presence of EV strains of different serotypes and their diversity between sewage and clinical samples obtained in the United Kingdom, Pakistan, and Senegal, providing a more detailed picture of EV circulation and showing the value of this approach to detect EV strains of public health relevance.

## METHODS

### Reference Viruses

Reference EV strains were used as controls. These included coxsackievirus (CV), echovirus (E), and EV strains: CV-B5 Faulkner, E-3 Morrisey, E-7 Wallace, E-20 JV-1, EV-D68 ATCC-VR 1197, and EV-A71 C4/523-07T. CV-A16 and CV-B4 isolates available at the National Institute for Biological Standards and Control (NIBSC) were also used.

### Sewage Sample Collection and Processing

Concentrates from sewage samples collected in Glasgow (Scotland, UK), London (England, UK), Gadap (Karachi, Pakistan), and Dakar (Senegal) were used in this study (Supplementary Table 1). One-liter raw sewage specimens were collected using the grab method, except for London, where composite samples were collected during a 24-hour period. Samples were processed using the 2-phase separation method following World Health Organization (WHO) Guidelines [11].

### Virus Isolation in Cell Culture

Virus isolation in cell cultures was performed according to WHO recommendations [22]. For each sample, 0.5-mL aliquots of sewage concentrate were inoculated on rhabdomyosarcoma (RD) 25-cm^2^ cell culture flasks. Cells showing full CPE were collected and stored at <–20°C for further processing.

### Identification by NGS Analysis of EV Strains Present in Sewage Concentrates

Full details of the preparation of Pan-EV entire capsid-coding region (ECRA) RT-PCR templates and subsequent NGS analysis are available in the Supplementary Data section. Raw fastq NGS files are available from NCBI’s Sequence Read Archive under project code PRJNA436746.

### Quantification of EV Viral RNA in Sewage Concentrates by RT-qPCR

The EV RNA content in sewage concentrates was estimated by real-time RT-PCR using a qScript XLT qPCR Toughmix system (Quantabio) in a Rotor-Gene Q instrument (Qiagen) and a 2-step protocol including a reverse transcription (RT) step and a complementary DNA (cDNA)–based qPCR step. Details of primers, amplification methods, and quantification analysis are provided in the Supplementary Data section.

### Nucleotide Sequence Analysis of the VP1 Coding Region of EV Strains by the Sanger Method

VP1 nucleotide sequences of relevant EV strains identified by NGS were also analyzed by Sanger sequencing. PCR fragments containing partial/full VP1 sequences were generated from purified Pan-EV RT-PCR products by PCR using a Platinum Taq High Fidelity Enzyme (Invitrogen) system and primers specific to the serotype of interest (primer sequences available on request). Amplified products were purified using the QIAquick PCR purification kit (Qiagen) and sequenced using an ABI Prism 3130 genetic analyzer (Applied Biosystems). Nucleotide sequences are available from GenBank with accession numbers MH084293–MH084344.

### Phylogenetic Analysis of EV Strains

EV sequences obtained in this study were compared with those of other EVs available in the GenBank database. EV genome sequences were aligned using the program ClustalW (within Geneious). The Molecular Evolutionary Genetics Analysis (MEGA) software package, version 7.0 [23], was used for phylogenetic analyses, as described in Figures 2 and 3.

### Comparison of VP1 Sequences of EV Strains From ES and Clinical Samples

VP1 sequences from EV strains found in the sewage samples collected in Senegal and Pakistan were compared with VP1 sequences of cultured EV isolates obtained from acute flaccid paralysis (AFP) cases in West Africa during 2013–2014 [24] and a small subset of 2013 AFP cases from Pakistan [25–27], respectively. In addition, VP1 sequences from EV sewage strains from Glasgow and London were compared with VP1 sequences from samples (mostly cerebrospinal fluid but a number from throat swabs and feces) of 2015 meningitis cases in Scotland obtained by direct sequencing of RT-PCR products [28].

## RESULTS

### Identification by NGS of EV Strains Present in Sewage Concentrates

We first analyzed virus reference samples of known nucleotide sequences using laboratory mixtures. Four control samples containing EV species A, B, C, and D strains in different combinations/concentrations were used. NGS reads were filtered and analyzed as described in the Supplementary Data section. The results shown in Supplementary Figure 1 and Supplementary Table 2 demonstrate our ability to accurately identify sequences of individual EV strains in mixtures; in all control samples analyzed, nucleotide sequences determined by NGS of EV strains present in mixtures were identical or almost identical (99.9%–100%) to the known sequences of individual viruses.

**Figure 1.**
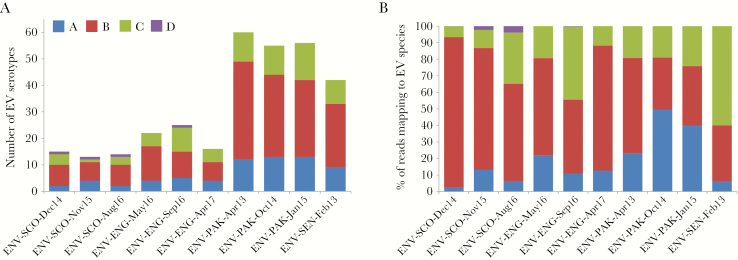
Enterovirus (EV) species and serotype composition in sewage samples. The number of EV serotypes detected in each sewage sample (A) and the percentage of next-generation sequencing reads mapping to EV strains from each of the 4 different EV species (B) are shown. EV species A (blue), B (red), C (green), and D (purple).

We then followed the same analytical process using NGS data obtained from Pan-EV RT-PCR products amplified from viral RNAs purified from sewage concentrates. A high proportion of the total filtered NGS reads from all samples mapped to final EV contig consensus sequences (range, 93.2%–98.7%; median, 97.4%), which shows the high specificity of the primers used for EV sequences and the high proportion of full-capsid sequences present in RT-PCR products. A large variety of EV serotypes were found among the 10 sewage samples analyzed, with EV strains of as many as 93 different serotypes identified in total. As shown in Figure 1 and Supplementary Table 3, sewage concentrates from Pakistan contained the largest number of serotypes per sample (55–60) and a total of 86 different serotypes, 42 different serotypes were identified in the sample from Senegal, and sewage concentrates from the United Kingdom had a lower serotype diversity (13–25 per sample and a total of 42 different serotypes in the 6 samples tested). In addition, sewage samples were found to contain between 5.1 and 7.5 log_10_ EV genome copies/L by RT-qPCR, with samples from the United Kingdom, Pakistan, and Senegal showing an average of 322, 18 995, and 14 088 log_10_ EV genome copies per mL, respectively. The detailed EV serotype composition for each sample is shown in Supplementary Table 3, which depicts the proportion of filtered NGS reads mapping to each of the identified serotypes. The prevalence and relative proportions of EV serotypes were different between sewage samples, but sequential samples from the same location contained a large number of genetically related strains from many serotypes (18 in the United Kingdom and 42 in Pakistan), some being almost identical. In addition, EV strains from serotypes EV-A76, EV-A89, and CV-A1 from Pakistan were found to be genetically close to UK sewage strains (>98.0% VP1 sequence identity) and possibly the result of importation (Supplementary Table 3).

The presence of viable EVs in sewage samples was shown by virus isolation on RD cells. Capsid sequences of these virus isolates were highly similar (>99% VP1 sequence identity) to those of the corresponding sewage EV strains (data not shown). EV strains from only a few serotypes were found in infected RD cultures. They included coxsackievirus B (B1 to B5) and echovirus (3, 6, 7, 11, 13, 19, and 20) strains, as well as PV2 and PV3 found in a sample from Pakistan and England, respectively.

Several negative control samples were sequenced, including RNA extraction, RT-PCR reaction, and water controls for each NGS run and cell culture media and uninfected RD cell culture extracts. Although few sequence reads mapping to EV sequences were found in some of these samples, as typically found in MiSeq-negative controls [29], the depth of coverage and length of contigs were well below the limits set up for contig selection.

### Genetic Relationship Between EV Strains From Sewage and Clinical Samples

VP1 sequences from EV strains identified in sewage concentrates were compared with VP1 sequences from contemporary clinical isolates. Given the rapid evolution of EVs during human-to-human transmission, accumulating mutations in the range of 0.41 × 10^−2^ to 3.07 × 10^−2^ substitutions/site/year [30], EVs have the potential to rapidly generate high genetic diversity when spreading to different geographic locations. Hence, we have considered a VP1 nucleotide sequence identity >95% as an indication of EV strains from different locations being genetically related. Based on this assumption, numerous EV strains genetically related to contemporary clinical isolates from the same geographical region were found in the sewage samples from the United Kingdom, Pakistan, and Senegal. Table 1 shows those with >97.5% VP1 nucleotide sequence identity.

**Table 1.** Genetic Relationships Between VP1 Sanger Sequences of EVs From Clinical Samples^a^ and VP1 NGS Sequences of EV Strains Identified in Sewage Samples

Sewage Sample	Serotype	Clinical Isolate (Accession No.)	Country	Year	% Identity
ENV-SEN-Feb13	CV-B2	KY433601	Senegal	2013	99.3
ENV-SEN-Feb13	CV-B2	KY433603	Senegal	2014	98.2
ENV-SEN-Feb13	CV-B4	KY433740	Mauritania	2013	99.5
ENV-SEN-Feb13	CV-B4	KY433737	Guinea	2013	98.8
ENV-SEN-Feb13	E-7	KY433617	Senegal	2013	98.7
ENV-SEN-Feb13	E-11	KY433626	Senegal	2013	98.7
ENV-SEN-Feb13	E-13	KY433642	Senegal	2014	97.8
ENV-SEN-Feb13	E-24	KY433666	Gambia	2013	99.1
ENV-SEN-Feb13	E-24	KY433665	Guinea	2013	99.0
ENV-SEN-Feb13	E-24	KY433663	Senegal	2013	98.4
ENV-SEN-Feb13	E-25	KY433722	Senegal	2014	98.6
ENV-SEN-Feb13	E-25	KY433723	Guinea-B	2014	97.7
ENV-SEN-Feb13	E-29	KY433782	Senegal	2014	98.7
ENV-SEN-Feb13	E-30	KY433671	Senegal	2013	99.2
ENV-SEN-Feb13	E-30	KY433673	Senegal	2014	97.9
ENV-SEN-Feb13	E-33	KY433679	Senegal	2013	99.9
ENV-ENG-May16	CV-A4^b^	This paper	Scotland	2015	97.7
ENV-SCO-Nov15	CV-A6^b^	This paper	Scotland	2015	99.1
ENV-SCO-Dec14	CV-A9	This paper	Scotland	2015	97.9
ENV-SCO-Dec14	CV-B2	This paper	Scotland	2015	97.8
ENV-SCO-Aug16	CV-B2	This paper	Scotland	2015	99.3
ENV-ENG-May16	CV-B2	This paper	Scotland	2015	98.9
ENV-ENG-Sep16	CV-B2	This paper	Scotland	2015	99.2
ENV-SCO-Dec14	CV-B4	This paper	Scotland	2015	99.9
ENV-SCO-Nov15	CV-B5	This paper	Scotland	2015	99.9
ENV-ENG-May16	CV-B5	This paper	Scotland	2015	98.9
ENV-ENG-Apr17	CV-B5	This paper	Scotland	2015	98.9
ENV-SCO-Nov15	E-6	This paper	Scotland	2015	99.9
ENV-ENG-May16	E-6	This paper	Scotland	2015	99.1
ENV-SCO-Dec14	E-9	This paper	Scotland	2015	99.6
ENV-ENG-Apr17	E-9	This paper	Scotland	2015	97.9
ENV-ENG-May16	E-11	This paper	Scotland	2015	97.8
ENV-SCO-Dec14	E-30	This paper	Scotland	2015	99.8
ENV-PAK-Apr13	E-19	KP814121	Pakistan	2013	99.2
ENV-PAK-Apr13	E-19	KP814124	Pakistan	2013	99.1
ENV-PAK-Apr13	E-19	KP814131	Pakistan	2013	98.7
ENV-PAK-Oct14	CV-B5	KY593471	Pakistan	2014	98.2
ENV-PAK-Oct14	CV-B5	KY593467	Pakistan	2013	98.7
ENV-PAK-Apr13	EV-B77	KM486571	Pakistan	2013	97.9

Abbreviations: AFP, acute flaccid paralysis; EV, enterovirus; NGS, next-generation sequencing.

^a^Cultured isolates from AFP samples from Senegal and Pakistan and meningitis samples from Scotland.

^b^VP4 sequences.

### Genetic Characterization of Selected PV, EV-A71, CV-A16, and EV-D68 Strains Found in Sewage Concentrates

The nucleotide sequences of several EV strains from relevant serotypes were further analyzed. PV type 2 and type 3 strains were identified in sewage samples from Pakistan (April 2013) and England (September 2016), respectively (Supplementary Table 3). The PV2 sewage strain from Pakistan was identified as a type 2 vaccine-derived PV showing a 2.9% VP1 sequence drift from Sabin 2 and as a PV recombinant with a crossover point between nucleotides 3649 and 3663 as sequences from this point were genetically distant to Sabin 2 (79.3% VP1 nucleotide sequence identity). The PV3 sewage strain from London was characterized as a vaccine-like PV with only 1 nucleotide mutation found at nucleotide 2493.

**Figure 2.**
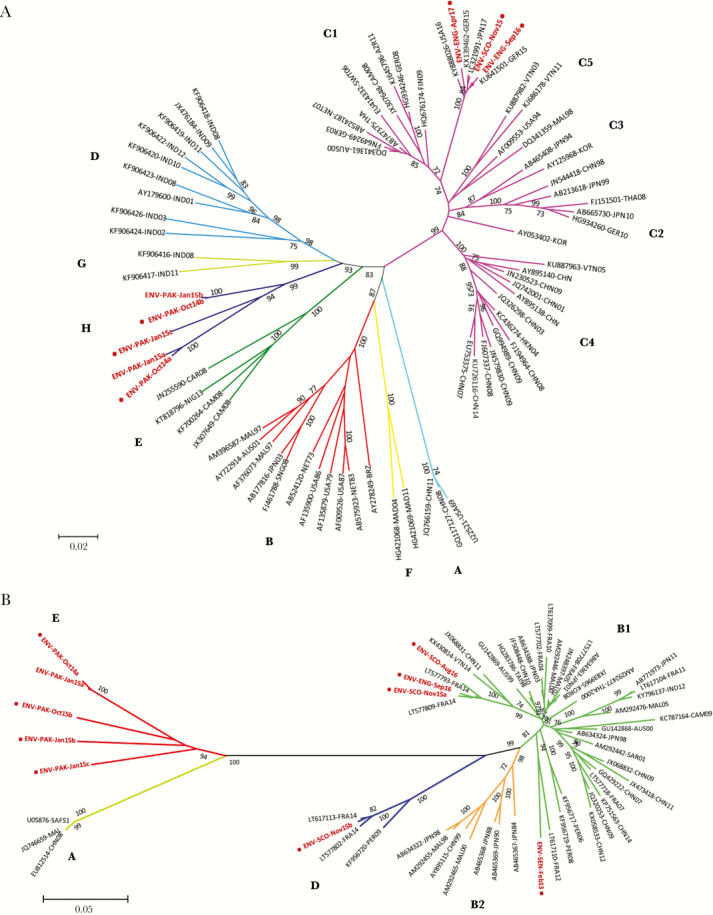
Phylogenetic analysis of VP1 sequences of EV-A71 and CV-A16 strains found in sewage samples. Phylogenetic analyses were conducted in MEGA7 [23]. The evolutionary history for EV-71 (A) and CV-A16 (B) strains found in sewage samples was inferred using the neighbor joining method. The optimal tree with a sum of branch length = 2.8367 (EV-A71) or 1.7168 (CV-A16) is shown. The unrooted tree is drawn to scale, with branch lengths in the same units as those of the evolutionary distances used to infer the phylogenetic tree. The evolutionary distances were computed using the maximum composite likelihood method and are in the units of the number of base substitutions per site. The GenBank accession number, country of origin, and isolation date are indicated in the name of each reference sequence used in the analysis. Numbers at nodes indicate the percentage of 1000 bootstrap pseudoreplicates supporting the cluster. Genogroups A–H (EV-A71) and A–D (CV-A16) are indicated next to clustered sequences. Names for phylogenetic groups (genogroups and subgenogroups) were assigned as in Bessaud et al. [39] for EV-A71 and as in Hassel et al. [5] for CV-A16. VP1 sequences from this study, determined by next-generation sequencing, are shown in red. A red dot next to a sequence name indicates that this sequence was also determined by the Sanger method. Abbreviations: USA, United States; CAR, Central African Republic; BRZ, Brazil; NIG, Nigeria; CAM, Cameroon; MAL, Malaysia; AUS, Australia; SNG, Singapore; MAD, Madagascar; NET, Netherland; IND, India; JPN, Japan; CHN, China; THA, Thailand; GER, Germany; NZL, New Zealand; TAI, Taiwan; FIN, Finland; AZR, Azerbaijan; VTN, Vietnam; KOR, South Korea; FRA, France; HKN, Hong Kong; SWT, Switzerland; SAF, South Africa; SEN, Senegal; PER, Peru; SAR, Saudi Arabia.

New EV-A71 and CV-A16 genogroups were identified for the first time in Pakistan. The UK EV-A71 strains were closely related to EV-A71 clinical isolates from Europe, the United States, and Japan, but all EV-A71 sewage strains from Pakistan grouped together in a separate branch of the phylogenetic tree in what appears to be a new genogroup that we have named H (Figure 2A), genetically distant (<85% VP1 sequence identity) to all other known genogroups. These EV-71 strains from Pakistan showed high genetic variability between them (89.6%–99.6% VP1 sequence identity) but shared similar amino acid sequences (98.3%–100%). They were genetically closer to strains from genogroups D and G from India (84.9% and 85.0% average VP1 sequence identity, respectively) [31] and showed high amino acid similarity with them (98.3%–100% in VP1). CV-A16 strains were identified in 2 sewage samples from Scotland, 1 from England, 2 from Pakistan, and the sample from Senegal (Supplementary Table 3). As shown in Figure 2B, the UK CV-A16 strains were closely related to clinical isolates from France. The CV-A16 sewage strain from Senegal clustered within genogroup B but only had 91.5% VP1 sequence identity with its closest relative (a 2012 isolate from France). All CV-A16 sequences from Pakistan clustered together in a separate branch of the phylogenetic tree in what appears to be a new genogroup that we have named E (Figure 2B). Genogroup E strains were genetically distant (<78.5% VP1 nucleotide sequence identity) to all known CV-A16 isolates except the first 1 reported from South Africa in 1951 (Acc. No. U05876). Remarkably, the CV-A16 sewage strains from Pakistan were genetically closer to this isolate (82.7%–84.7% VP1 sequence identity) and showed high amino acid similarity (98.3%–99.3% in VP1) with it despite their 63-year “age” difference. The CV-A16 strains from Pakistan showed high genetic variability between them (86.8%–99.1% VP1 sequence identity) but shared very similar amino acid sequences (99.3%–100% in VP1).

**Figure 3.**
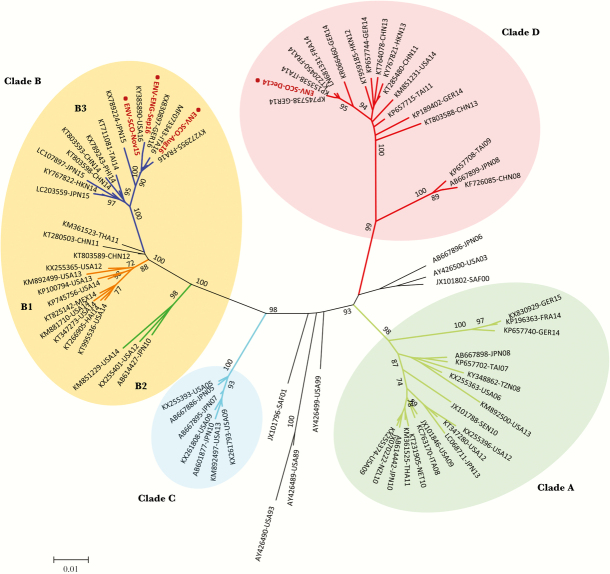
Phylogenetic analysis of VP1 sequences of EV-D68 strains found in sewage samples. Phylogenetic analyses were conducted in MEGA7 [23]. The evolutionary history for EV-D68 strains found in sewage samples was inferred using the neighbor joining method. The optimal tree with a sum of branch length = 0.9034 is shown. The unrooted tree is drawn to scale, with branch lengths in the same units as those of the evolutionary distances used to infer the phylogenetic tree. The evolutionary distances were computed using the maximum composite likelihood method and are in the units of the number of base substitutions per site. The GenBank accession number, country of origin, and isolation date are indicated in the name of each reference sequence used in the analysis. Evolutionary analyses were conducted in MEGA7 [23]. Numbers at nodes indicate the percentage of 1000 bootstrap pseudoreplicates supporting the cluster. Genetic clades A to D are indicated next to clustered sequences. Names for phylogenetic groups (genetic clades) were assigned as in Gong et al. [40]. VP1 sequences from this study, determined by next-generation sequencing and Sanger, are shown in red. Abbreviations: ITA, Italy; USA, United States; MEX, Mexico; JPN, Japan; CHN, China; THA, Thailand; GER, Germany; SAF, South Africa; NZL, New Zealand; TAI, Taiwan; NET, Netherlands; SEN, Senegal; TZN, Tanzania; FRA, France; HAI, Haiti; HKN, Hong Kong; NZL, New Zealand; SEN, Senegal; SAF, South Africa, PHI; Philippines.

EV-D68 strains were only found in sewage samples from the United Kingdom, the 3 samples from Scotland (December 2014, November 2015, and August 2016), and 1 sample from England (September 2016) (Supplementary Table 3). As shown in Figure 3, the UK D68 sewage strains were very closely related to epidemic strains from both the 2014 and 2016 EV-D68 outbreaks that spread worldwide.

Some sewage EV strains identified corresponded to uncommon serotypes, defined as those rarely associated with clinical cases and/or hardly ever reported elsewhere in the world. Table 2 shows the genetic properties of some of these strains in comparison to their closest relatives from the GenBank database. Most of these “rare” serotypes were identified in the sewage samples from Pakistan (13/14), some in the sample from Senegal (4/14), and a few in 2 samples from the United Kingdom (2/14).

**Table 2.** Genetic Properties of Uncommon EV Strains Found in Sewage Concentrates

EV Serotype	Sample ID	Closest Relative From GenBank Sequence Database				
		Accession No.	Nucleotide Identity, %^a^	Amino Acid Identity, %^a^	Country	Date
A114	ENV-PAK-Apr13	KU355876	82.6	97.6	India	2013
	ENV-PAK-Oct-14	KU355876	83.0	97.6	India	2013
A119	ENV-SEN-Feb13	KT285372	95.2	99.7	Ivory Coast	2013
A120	ENV-PAK-Apr13	KF700245	82.6	98.0	Tajikistan	2013
	ENV-SEN-Feb13	KF700245	92.2	98.6	Tajikistan	2013
A121	ENV-PAK-Jan15	KU355877	95.8	99.0	India	2013
	ENV-PAK-Oct14	KU355877	95.3	99.0	India	2013
B78	ENV-PAK-Jan15	JN204068	86.8	97.7	India	2008
	ENV-PAK-Oct14	JN204068	86.4	97.3	India	2008
	ENV-ENG-May16	JN204070	90.4	98.5	India	2008
B88	ENV-PAK-Jan15	JN204092	83.7	94.8	India	2008
	ENV-PAK-Apr13	JN204092	83.9	95.5	India	2008
	ENV-PAK-Oct14	JN204092	83.1	95.5	India	2008
B100	ENV-PAK-Oct14	KF453626	96.1	97.9	Pakistan	2010
B101	ENV-PAK-Jan15	AY843308	82.8	96.7	Ivory Coast	2002
	ENV-PAK-Oct14	AY843308	82.4	96.7	Ivory Coast	2002
B106	ENV-PAK-Jan15	KF385945	95.3	97.6	Pakistan	2010
	ENV-PAK-Apr13	KF385945	94.2	96.9	Pakistan	2010
	ENV-PAK-Oct14	KF385945	93.6	96.2	Pakistan	2010
	ENV-SEN-Feb13	KX171334	82.6	93.8	China	May-11
B107	ENV-PAK-Jan15	KR065422	92.5	98.6	Pakistan	2009
	ENV-PAK-Apr13	KR065422	92.4	98.6	Pakistan	2009
EV-C95	ENV-PAK-Jan15	KM273014	81.2	97.3	Djibouti	2003
EV-C102	ENV-PAK-Jan15	EF555645	83.1	96.7	Bangladesh	1999
EV-C113	ENV-PAK-Jan15	KC344833	94.8	99.3	Bangladesh	2006
EV-C116	ENV-PAK-Jan15	JX514942	95.3	98.0	Russia	2010
	ENV-PAK-Oct14	JX514942	95.8	98.7	Russia	2010
	ENV-SEN-Feb13	KT285371	94.4	100	Ivory Coast	2013
	ENV-ENG-May16	JX514942	96.3	98.7	Russia	2010
	ENV-ENG-Sep16	JX514942	96.3	98.7	Russia	2010

Abbreviation: EV, enterovirus.

^a^VP1 sequences compared.

VP1 nucleotide sequences of all EV strains described in this section were also determined by the Sanger method using specific primers designed for this study. In all cases, there was perfect or nearly perfect agreement (99.8%–100%) between the NGS and Sanger sequencing data.

## DISCUSSION

Here we describe the direct detection and characterization of EV strains present in sewage samples using a cell culture–free NGS approach. Many EV strains of all 4 A, B, C, and D species and 93 different serotypes were identified in a total of 10 sewage samples from the United Kingdom, Senegal, and Pakistan. Sewage concentrates from Pakistan and Senegal contained a higher number of serotypes per sample than those from the United Kingdom. This agrees with the fact that EV infections are more common in developing countries [2]; however, other factors such as human population movements, climate, and sampling site characteristics are likely to influence the diversity and type of viruses that can be found in sewage. Not surprisingly, the sewage concentrates from the United Kingdom contained lower EV concentrations (5.1–5.6 log_10_ EV genome copies/L) than those present in Senegal and Pakistan concentrates (7.0–7.5 log_10_ EV genome copies/L), which are at the high end of the range of what has been reported before [32]. All sewage samples from the United Kingdom and 1 sample from Pakistan contained a higher proportion of EV-B sequences, the other 2 samples from Pakistan had a higher concentration of EV-A genomes, and the sample from Senegal showed a higher percentage of EV-C sequences. EV-D strains (all from serotype EV-D68) were only found in sewage samples from the United Kingdom. Studies using a variety of cell lines or direct molecular typing have shown the prevalence of EV-B strains in sewage in Europe and the United States (typically >75% of all EVs), with the occasional upsurge of EV-A and EV-D serotypes in recent years [15, 21]. Interestingly, a recent study in Scotland identified a high quantity of EV-C strains in sewage samples (95 amplicons from 353 cloned EV sequences, 26.9%) [14]. High frequency of EV-C serotypes has been reported in tropical countries, for example, 26.5% of all EV strains found in sewage samples from 2007–2013 in Senegal [12] and 39.5%–63.1% of EV isolates identified in clinical samples from 2008 in Cameroon [33], consistent with our results with the sample from Senegal (60.0% of EV-C sequences). Our results also confirmed the high sensitivity of RD cells for infection with species B EVs and PV strains, as only these viruses were found in RD cultures infected with sewage concentrates. This shows the great advantage of using a direct detection method as it does not only allow the identification of multiple unculturable EV strains but also prevents the presence of cell culture–induced mutations that would potentially be present in cell culture EV isolates.

The NGS results presented here were supported by extensive Sanger sequence analysis of VP1 nucleotide sequences from EV strains of different serotypes. In addition, identification of several EVs in sewage was shown to correlate with detection in clinical samples. Remarkably, a high proportion (64.7%–79.1%) of clinical isolates from the same or neighboring countries obtained during a 1–2-year period around the time of sewage collection [24–27] was represented in sewage samples from the United Kingdom, Pakistan, and Senegal, as EV strains genetically related to these EV clinical isolates were found in sewage concentrates. In addition, a large number of genetically related EV strains from many serotypes were found in sequential samples from each country (Supplementary Table 3), providing evidence of the widespread and sustained circulation of many EV serotypes in different geographical regions, some linked to clinical manifestations. This level of detail has never been described before in similar samples and opens new avenues for the molecular characterization of EV mixtures in clinical and environmental samples using NGS.

We found several EV strains of public health relevance, including a type 3 vaccine-like PV in a sample from London and a type 2 recombinant vaccine-derived PV in a sample from Pakistan. We also identified strains from serotypes that have been associated with large HFMD outbreaks in recent years, occasionally leading to neurological disease, including the first records of CV-A16 and EV-A71 serotypes in Pakistan, representing novel divergent genogroups indigenous to this country. The fact that severe complications associated with EV-A71 infections mostly occur in countries of the Asia-Pacific region is intriguing as EV-A71 appears to be widely circulating in other areas of the world. New genogroups have been recently described in India [31] and Africa [34], and also now in Pakistan, as we show here. It will be worth investigating further the differences in clinical severity induced by EV-A71 infections in different geographical regions. In addition to HFMD strains, several EV-D68 strains genetically linked to clinical isolates from the 2014 and 2016 outbreaks that spread worldwide were found in sewage samples from the United Kingdom. Notably, EV-D68 strains present in sewage samples collected in Scotland (November 2015 and August 2016) and England (September 2016) clustered within genetic subclade B3 and were very closely related to 2016 clinical isolates from Europe and the United States [6, 7]. The earliest of such clinical isolates was from April 2016 in Italy, 4 months after our first detection in sewage. This supports the idea that ES can contribute to the early detection of emerging EV serotypes and the assessment of the geographical and temporal spread of EV outbreaks.

Some of the EV strains identified in the sewage samples analyzed corresponded to rarely reported serotypes. These included serotypes A119 and B78, for which no full genome sequences are available, recently reported new serotypes such as A114, A120, A121, B106, and C113 [35–38], and other serotypes for which there have been only a few isolates reported and little genetic information is available.

In conclusion, we have identified and characterized many EV strains in sewage samples using a novel approach that involves NGS analysis of PanEV-specific PCR products obtained directly from sewage concentrates. This approach could help quickly expand the pool of nucleotide sequences of EVs available from different geographical regions, particularly from countries where clinical diagnostic data are poor or not available, and contribute to a better understanding of the circulation patterns of EVs in humans and their possible implication in human disease. PV was found in sewage samples, so this method could help the rapid identification of PV in environmental samples, which is critical to support the global polio eradication endgame.

## Supplementary Material

ofy250_suppl_supplementary_table_s1

ofy250_suppl_supplementary_table_s2

ofy250_suppl_supplementary_table_s3

ofy250_suppl_supplementary_fig_s1

ofy250_suppl_supplementary_fig_s2

ofy250_suppl_supplementary_data
