# Supplementary material for: Environmental Surveillance Reveals Complex Enterovirus Circulation Patterns in Human Populations
Source: Open Forum Infect Dis. 2018 Oct 1;5(10):ofy250. doi: 10.1093/ofid/ofy250 (PMC6201154; doi:10.1093/ofid/ofy250)
Supplement: ofy250_suppl_supplementary_table_s1 [file ofy250_suppl_supplementary_table_s1.docx]

| **SUPPLEMENTARY TABLE 1.** Details of sewage samples used in this study | | | |
| --- | --- | --- | --- |
| Location | Sample ID | Type of sample | Collection Date |
| London (England, UK) | ENV-ENG-May16 | 24h composite | May-2016 |
|  | ENV-ENG-Sep16 | 24h composite | Sep-2016 |
|  | ENV-ENG-Apr17 | 24h composite | Apr-2017 |
| Glasgow (Scotland, UK) | ENV-SCO-Dec14 | Grab | Dec-2014 |
|  | ENV-SCO-Nov15 | Grab | Nov-2015 |
|  | ENV-SCO-Aug16 | Grab | Aug-2016 |
| Gadap (Karachi, Pakistan) | ENV-PAK-Apr13 | Grab | Apr-2013 |
|  | ENV-PAK-Oct14 | Grab | Oct-2014 |
|  | ENV-PAK-Jan15 | Grab | Jan-2015 |
| Dakar (Senegal) | ENV-SEN-Feb13 | Grab | Feb-2013 |
