# Supplementary material for: Environmental Surveillance Reveals Complex Enterovirus Circulation Patterns in Human Populations
Source: Open Forum Infect Dis. 2018 Oct 1;5(10):ofy250. doi: 10.1093/ofid/ofy250 (PMC6201154; doi:10.1093/ofid/ofy250)
Supplement: ofy250_suppl_supplementary_table_s2 [file ofy250_suppl_supplementary_table_s2.docx]

| **SUPPLEMENTARY TABLE 2.** Genetic identity of EV strains identified in control samples. | | | | |
| --- | --- | --- | --- | --- |
| Sample | Virus strain | Coverage (nt position) | NCBI ID | Identity (%) |
| Control 1 | CV-A71 C4/523-07T | 560-4416 | This paper | 100 |
|  | E-7 Wallace | 557-4405 | AY036579 | 100 |
|  | PV-1 Sabin | 553-4459 | AY184219 | 100 |
|  | EV-D68 ATCC-VR­1197 | 535-4365 | KT725431 | 100 |
| Control 2 | CV-B5 Faulkner | 558-4378 | AF114383 | 100 |
|  | E-7 Wallace | 557-4356 | AY036579 | 100 |
|  | E-20 JV-1 | 581-4372 | AY302546 | 100 |
|  | PV-1 Sabin | 553-4459 | AY184219 | 99.9 |
| Control 3 | CV-A16 NIBSC | 580-4432 | This paper | 100 |
|  | CV-B4 NIBSC | 558-4373 | This paper | 99.9 |
|  | E-3 Morrisey | 558-4406 | AY302553 | 99.9 |
|  | E-7 Wallace | 581-4405 | AY302559 | 100 |
| Control 4 | PV-1 Sabin | 557-4487 | AY184219 | 99.9 |
|  | PV-2 Sabin | 535-4467 | AY184220 | 99.9 |
|  | PV-3 Sabin | 541-4467 | AY184221 | 100 |
