# Supplementary material for: Environmental Surveillance Reveals Complex Enterovirus Circulation Patterns in Human Populations
Source: Open Forum Infect Dis. 2018 Oct 1;5(10):ofy250. doi: 10.1093/ofid/ofy250 (PMC6201154; doi:10.1093/ofid/ofy250)
Supplement: ofy250_suppl_supplementary_table_s3 [file ofy250_suppl_supplementary_table_s3.docx]

| **SUPPLEMENTARY TABLE 3.** EV serotype composition in sewage samples from different locations^1^. | | | | | | | | | | | | |
| --- | --- | --- | --- | --- | --- | --- | --- | --- | --- | --- | --- | --- |
| Species/Serotype | | Scotland | | | England | | | Pakistan | | | Senegal | |
|  |  | Dec-14 | Nov-15 | Aug-16 | May-16 | Sep-16 | Apr-17 | Apr-13 | Oct-14 | Jan-15 | Feb-13 |  |
| A | CV-A2 |  |  |  |  |  |  |  |  | 1.2 |  |  |
|  | CV-A3 |  |  |  |  |  |  | 0.4 |  |  | 0.3 |  |
|  | CV-A4 |  | 3.2 | 0.8 | 9.9^2^ | 4.2 |  | 0.4 |  | 0.7 |  |  |
|  | CV-A5 |  |  |  |  |  | 6.4 | 0.5 | 0.6 | 0.2 | 1.4 |  |
|  | CV-A6 | 0.7 | 4.9 |  | 5.9 |  |  |  | 1.0 | 2.4 | 1.0 |  |
|  | CV-A7 |  |  |  |  |  |  |  |  |  | 0.2 |  |
|  | CV-A8 |  |  |  |  |  |  | 0.2 |  | 1.1 |  |  |
|  | CV-A10 |  |  |  | 6.2 |  |  | 0.9 | 0.5 | 3.5 |  |  |
|  | CV-A12 |  |  |  |  |  |  | 1.2 | 0.5 |  |  |  |
|  | CV-A14 |  |  |  |  |  | 0.9 |  | 0.8 | 0.8 | 1.3 |  |
|  | CV-A16 |  | 4.4 | 5.7 |  | 1.2 |  |  | 0.7 | 0.8 | 0.3 |  |
|  | EV-A71 |  | 0.8 |  |  | 0.5 | 3.4 |  | 0.9 | 0.7 |  |  |
|  | EV-A76 | 1.7 |  |  |  | 3.4 | 1.9 | 4.2 | 18.5 | 22.2 |  |  |
|  | EV-A89 |  |  |  |  | 1.6 |  | 0.7 | 0.7 | 0.8 |  |  |
|  | EV-A90 |  |  |  |  |  |  | 2.9 | 7.0 | 2.0 | 0.8 |  |
|  | EV-A91 |  |  |  |  |  |  | 4.0 | 3.5 |  |  |  |
|  | EV-A114 |  |  |  |  |  |  | 0.1 | 0.4 |  |  |  |
|  | EV-A119 |  |  |  |  |  |  |  |  |  | 0.7 |  |
|  | EV-A120 |  |  |  |  |  |  | 0.5 |  |  | 0.4 |  |
|  | EV-A121 |  |  |  |  |  |  |  | 14.2 | 3.3 |  |  |
| B | CV-A9 | 10.2 |  | 14.6 |  |  |  | 0.4 |  |  |  |  |
|  | CV-B1 |  |  |  | 9.0 |  |  | 2.4 |  | 0.9 |  |  |
|  | CV-B2 | 2.3 | 5.8 | 12.4 | 5.4 | 8.3 |  |  |  |  | 1.4 |  |
|  | CV-B3 |  | 0.8 | 4.6 | 5.6 | 2.1 |  | 0.6 |  | 0.1 |  |  |
|  | CV-B4 | 42.5 |  |  | 2.7 | 9.8 | 5.8 |  |  |  | 5.0 |  |
|  | CV-B5 |  | 17.3 |  | 0.3 | 3.4 | 4.7 | 0.9 | 2.2 |  |  |  |
|  | CV-B6 |  |  |  |  |  |  |  | 0.2 |  |  |  |
|  | E-1 |  |  |  |  |  |  | 0.9 |  | 1.7 |  |  |
|  | E-2 |  |  |  |  |  |  | 0.5 | 0.2 |  | 0.8 |  |
|  | E-3 |  |  | 15.3 | 2.8 | 4.4 |  | 2.2 |  | 1.9 |  |  |
|  | E-4 |  |  |  |  |  |  |  | 1.4 | 0.8 |  |  |
|  | E-5 |  |  |  | 6.1 |  |  | 0.5 |  |  | 0.3 |  |
|  | E-6 | 7.5 | 31.8 |  | 3.4 | 2.7 | 3.9 | 0.6 | 0.6 | 4.1 | 0.7 |  |
|  | E-7 |  |  |  |  |  |  | 0.5 | 1.2 |  | 3.4 |  |
|  | E-9 | 10.8 |  |  | 4.4 |  | 9.3 |  | 1.2 | 6.6 | 1.1 |  |
|  | E-11 | 3.8 | 10.3 | 1.7 | 5.1 |  |  |  | 2.9 | 0.5 | 3.5 |  |
|  | E-12 |  |  |  |  |  |  | 0.3 |  |  |  |  |
|  | E-13 |  | 3.8 | 3.3 |  | 0.6 |  | 8.8 | 1.9 | 1.1 | 1.2 |  |
|  | E-14 |  |  |  |  | 7.1 | 8.1 | 1.2 | 0.2 | 2.9 | 0.8 |  |
|  | E-15 |  |  |  |  |  |  |  | 0.7 |  | 3.0 |  |
|  | E-17 |  |  |  |  |  |  | 3.0 | 1.5 | 3.5 | 0.4 |  |
|  | E-18 |  |  |  | 4.0 |  |  | 3.8 |  |  | 2.4 |  |
|  | E-19 |  |  |  |  | 2.7 |  | 6.1 |  | 0.2 |  |  |
|  | E-20 |  |  | 2.8 |  |  |  | 3.6 | 0.9 | 0.7 |  |  |
|  | E-21 |  |  |  |  | 3.5 |  | 1.4 |  |  | 0.6 |  |
|  | E-24 |  |  |  |  |  |  | 0.4 | 0.4 |  | 0.6 |  |
|  | E-25 | 8.6 | 3.7 | 3.8 |  |  | 1.0 | 1.2 | 1.5 |  | 1.2 |  |
| ^1^Heatmap showing percentage of NGS reads mapping to EV serotypes from highest (green) to lowest (red). Underlined figures indicate strain genetically linked to that in previous and/or next sample. | | | | | | | | | | | | |

| **SUPPLEMENTARY TABLE 3 contd.** EV serotype composition in sewage samples from different locations^1^. | | | | | | | | | | | |
| --- | --- | --- | --- | --- | --- | --- | --- | --- | --- | --- | --- |
| Species/Serotype | | Scotland | | | England | | | Pakistan | | | Senegal |
|  |  | Dec-14 | Nov-15 | Aug-16 | May-16 | Sep-16 | Apr-17 | Apr-13 | Oct-14 | Jan-15 | Feb-13 |
| B | E-26 |  |  |  |  |  |  | 1.1 |  |  |  |
|  | E-27 |  |  |  |  |  |  |  | 4.8^2^ | 1.1 |  |
|  | E-29 |  |  |  |  |  |  | 4.5 | 1.1 | 0.1 | 0.2 |
|  | E-30 | 5.3 |  |  | 6.7 |  | 42.7 | 2.7 |  | 0.3 | 1.0 |
|  | E-31 |  |  |  |  |  |  | 1.0 |  | 0.5 | 0.3 |
|  | E-32 |  |  |  |  |  |  | 0.4 | 0.7 | 1.2 |  |
|  | E-33 |  |  |  |  |  |  | 0.8 |  |  | 2.1 |
|  | EV-B69 |  |  |  |  |  |  |  | 0.5 |  |  |
|  | EV-B73 |  |  |  |  |  |  |  |  |  | 2.1 |
|  | EV-B74 |  |  |  |  |  |  | 2.0 | 0.4 | 0.3 |  |
|  | EV-B75 |  |  |  |  |  |  | 0.5 |  | 0.1 | 0.4 |
|  | EV-B77 |  |  |  |  |  |  | 1.1 |  |  |  |
|  | EV-B78 |  |  |  | 3.1 |  |  |  | 0.6 | 0.7 |  |
|  | EV-B79 |  |  |  |  |  |  | 1.6 |  |  |  |
|  | EV-B80 |  |  |  |  |  |  | 0.9 | 3.1 | 1.4 | 0.8 |
|  | EV-B81 |  |  |  |  |  |  |  | 0.4 | 0.3 |  |
|  | EV-B82 |  |  |  |  |  |  | 2.8 | 0.6 | 0.3 |  |
|  | EV-B83 |  |  |  |  |  |  | 0.4 |  |  |  |
|  | EV-B84 |  |  |  |  |  |  |  | 0.4 |  |  |
|  | EV-B85 |  |  |  |  |  |  |  | 0.7 |  |  |
|  | EV-B86 |  |  |  |  |  |  | 0.8 |  |  |  |
|  | EV-B88 |  |  |  |  |  |  | 0.2 | 0.1 | 0.8 |  |
|  | EV-B93 |  |  |  |  |  |  |  | 0.4 |  |  |
|  | EV-B97 |  |  |  |  |  |  |  |  | 1.4 |  |
|  | EV-B100 |  |  |  |  |  |  |  | 0.2 |  |  |
|  | EV-B101 |  |  |  |  |  |  |  | 0.1 | 1.2 |  |
|  | EV-B106 |  |  |  |  |  |  | 2.2 | 0.3 | 0.5 | 0.4 |
|  | EV-B107 |  |  |  |  |  |  | 0.8 |  | 0.8 |  |
| C | CV-A1 | 0.3 |  | 2.7 |  | 6.6 |  | 8.7 | 0.1 | 1.5 |  |
|  | CV-A11 |  |  |  | 1.5 | 1.8 |  | 0.4 | 0.1 | 1.0 | 0.3 |
|  | CV-A13 | 0.7 |  |  |  | 0.5 |  | 2.3 | 0.6 | 3.8 | 12.2 |
|  | CV-A17 |  |  |  |  |  | 5.3 | 1.6 | 1.9 | 3.8 | 1.1 |
|  | CV-A19 | 0.7 |  |  | 4.0 | 1.9 | 1.3 | 1.8 | 3.8 | 2.4 | 19.6 |
|  | CV-A20 |  |  |  |  |  | 1.8 | 0.2 | 0.5 | 0.7 | 7.1 |
|  | CV-A21 |  |  |  |  |  |  |  | 0.3 |  |  |
|  | CV-A22 | 4.8 | 11.0 | 27.9 | 11.0 | 26.5 |  | 0.7 |  | 2.0 | 0.3 |
|  | CV-A24 |  |  |  |  | 1.0 |  | 4.1 | 5.1 | 5.8 | 0.7 |
|  | EV-C95 |  |  |  |  |  |  |  |  | 0.4 |  |
|  | EV-C96 |  |  |  |  |  | 1.8 | 0.3 | 0.3 | 1.5 |  |
|  | EV-C99 |  |  | 0.5 | 0.9 | 0.5 | 1.5 | 0.8 | 1.8 | 0.2 | 13.4 |
|  | EV-C102 |  |  |  |  |  |  |  |  | 0.8 |  |
|  | EV-C113 |  |  |  |  |  |  |  |  | 0.1 |  |
|  | EV-C116 |  |  |  | 1.9 | 5.2 |  |  | 4.6 | 0.3 | 5.3 |
|  | PV-2 |  |  |  |  |  |  | 0.3 |  |  |  |
|  | PV-3 |  |  |  |  | 0.4 |  |  |  |  |  |
| D | EV-D68 | 0.1 | 2.3 | 3.9 |  | 0.3 |  |  |  |  |  |
| ^1^Heatmap showing percentage of NGS reads mapping to EV serotypes from highest (green) to lowest (red). Underlined figures indicate strain genetically linked to that in previous and/or next sample. | | | | | | | | | | | |
