# Supplementary figures and images for: Environmental Surveillance Reveals Complex Enterovirus Circulation Patterns in Human Populations
Source: Open Forum Infect Dis. 2018 Oct 1;5(10):ofy250. doi: 10.1093/ofid/ofy250 (PMC6201154; doi:10.1093/ofid/ofy250)

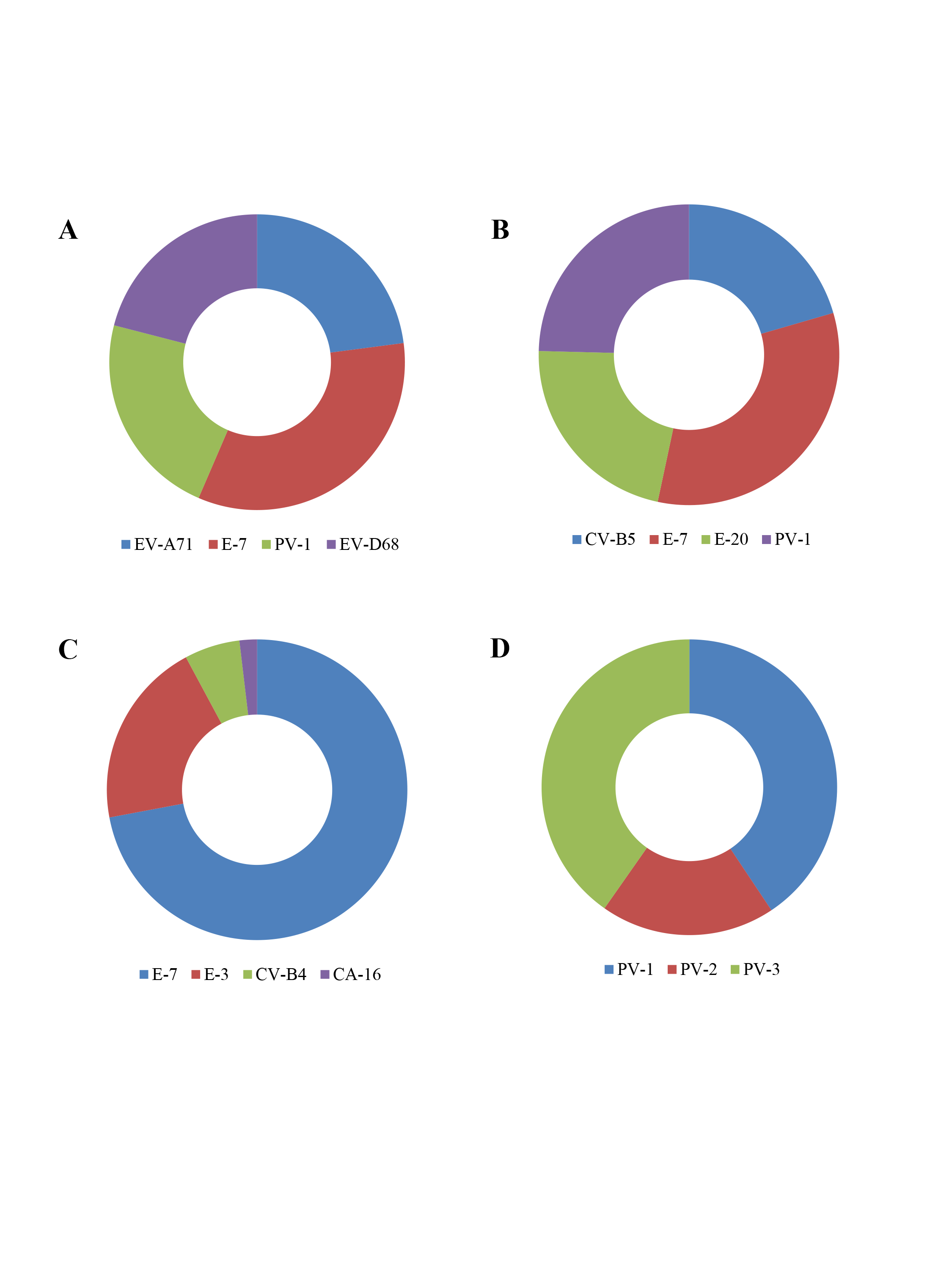

Supplement: ofy250_suppl_supplementary_fig_s1 [file ofy250_suppl_supplementary_fig_s1.png]

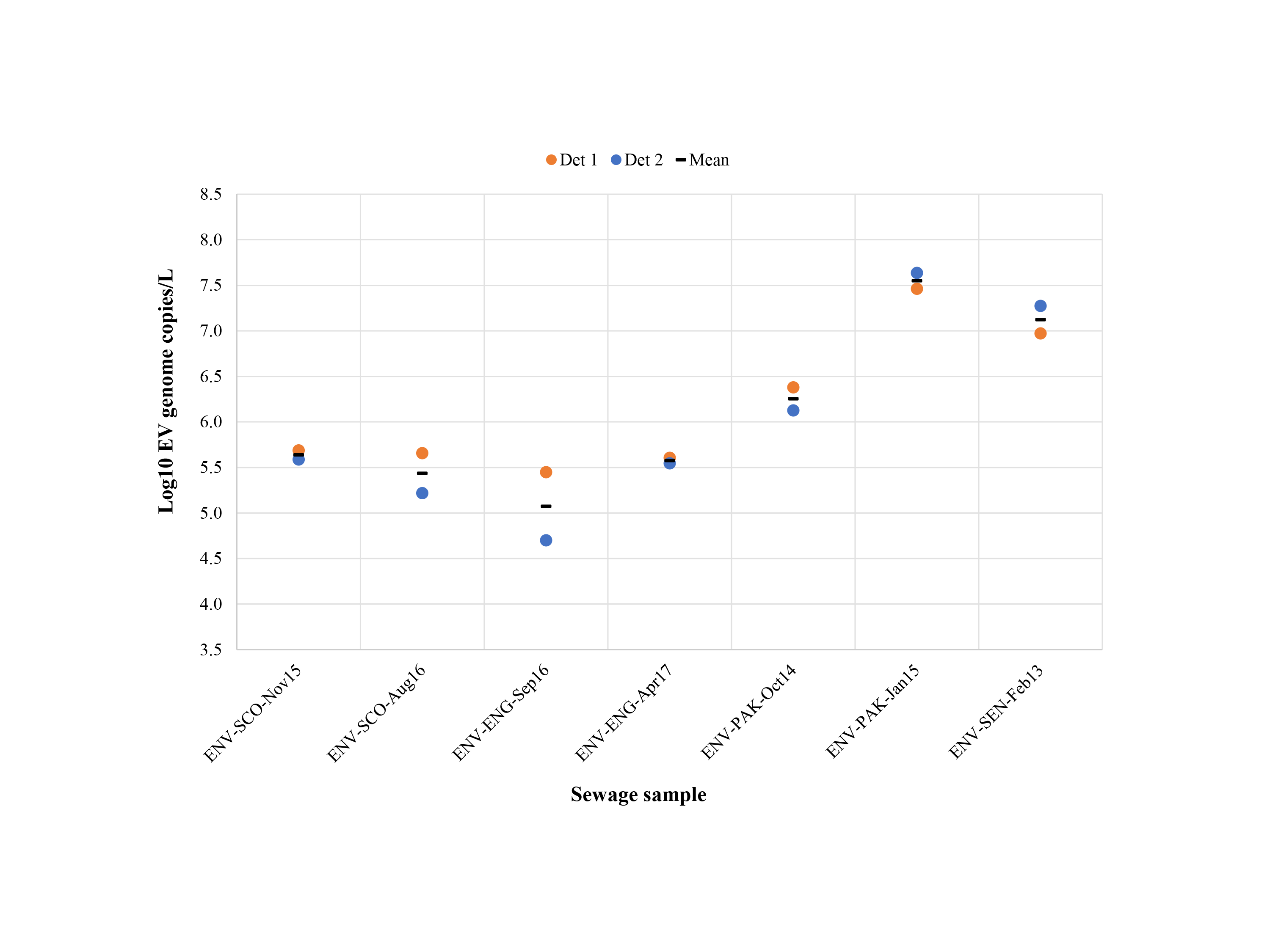

Supplement: ofy250_suppl_supplementary_fig_s2 [file ofy250_suppl_supplementary_fig_s2.png]
