# Supplementary material for: Environmental Surveillance Reveals Complex Enterovirus Circulation Patterns in Human Populations
Source: Open Forum Infect Dis. 2018 Oct 1;5(10):ofy250. doi: 10.1093/ofid/ofy250 (PMC6201154; doi:10.1093/ofid/ofy250)
Supplement: ofy250_suppl_supplementary_data [file ofy250_suppl_supplementary_data.docx]

**SUPPLEMENTARY DATA**

**METHODS**

**Preparation of Pan-EV entire capsid-coding region (ECRA) RT-PCR templates for NGS analysis**

We followed a modified version of the method described by Arita et al. [1]. using two independent reactions with two different primer sets that mapped in the same genomic regions (in different EV strains) producing amplicons approximately 3,900 nt in length with an aim to amplify sequences of EV strains from all four A, B, C and D species. The RT-PCR products covered the entire capsid-coding region and part of the region coding for non-structural proteins (2A-2C) (nucleotides 553-4459, numbering as in PV1 Sabin AY184219 reference strain). Viral RNA was purified from sewage concentrates and infected cell extracts using Roche High Pure viral RNA kit. RT-PCR fragments were amplified from purified viral RNAs by one-step RT-PCR using a SuperScript III One-Step RT-PCR System with Platinum Taq High Fidelity DNA Polymerase (Invitrogen). The two primer sets used were as follows:

a. Primers 5′NCR (5′-TGGCGGAACCGACTACTTTGGGTG-3′) and CRE-R (5′- TCAATACGGTGTTTGCTCTTGAACTG-3′)

b. Primers MM_EV_F2 (5’-CAGCGGAACCGACTACTTT-3’) and MM_EV_R1 (5′- AATACGGCATTTGGACTTGAACTGT-3′)

Amplification conditions were: 50 °C for 30 min followed by 94°C for 2 min plus 42 cycles of 94 °C for 15 s, 55 °C for 30 s and 68 °C for 8 min with a final extension step of 68 °C for 5 min. Amplified products from both reactions were purified using AMPure XP magnetic beads (Beckman Coulter) and pooled (1:1) before being sent for NGS analysis. We used 5-10 µl of total RNA purified from sewage concentrates in the RT-PCR reaction which corresponds to 1-2 ml of untreated raw sewage when considering the concentration factor following the sewage concentration and viral RNA extraction steps. Primers were tested using viral RNAs from EV-A, B, C and D control viruses and the RT-PCR reactions were found to detect at least 5-50 genome copies (data not shown), a similar sensitivity to that reported by Arita et al [1].

**Generation of sequencing libraries and quality trimming of NGS reads**

Sequencing libraries were prepared using Nextera XT reagents and sequenced on a MiSeq using a 2 x 301 paired-end v3 Flow Cell and manufacturer’s protocols (Illumina, California, USA). Raw sequence data were imported into Geneious R10 software (Biomatters) and paired end reads combined. Data were filtered using a custom workflow [2] with the following parameters: PCR primers and Nextera adaptor/index sequences were trimmed from 5′ and 3′ ends with a minimum 5 bp overlap; reads were trimmed to have no bases with a quality <Q30 and no ambiguities. Following this, reads <50 nt in length were discarded and duplicate reads were removed using the program Dedup (within Geneious). Raw fastq files are available from NCBI’s Sequence Read Archive (SRA) under project code PRJNA436746.

**Generation of EV sequence contigs by *de novo* assembly of filtered NGS reads**

The filtered NGS reads were assembled *de novo* using stringent assembly conditions as described before [2]: minimum 50 base overlap, minimum overlap identity of 98%, maximum 2% mismatches per read and only using paired hits during assembly. In addition, the options to produce scaffolds and ignore words repeated more than 100-1000 times, available in the Geneious assembler, were selected to improve the quality of assembly. Following BLAST analysis [3], only contigs with mean coverage >30nt per site and covering the entire capsid coding region were selected for further analysis. Filtered reads were finally mapped to these selected contigs to obtain final consensus sequences by assigning the most common nucleotide sequence to each nucleotide position. Manual analyses for visualizing and quantifying assembly results were performed throughout the process. As a result, we obtained nucleotide sequences for different EV strains in each sample. The closest virus relatives to each of the sewage EV final consensus sequences were identified using the RIVM and BLAST online sequence analysis tools [3, 4] and EV serotypes were assigned on the basis of their VP1 sequence.

**Quantification of EV RNA in sewage concentrates by RT-qPCR**

The quantity of EV RNA in sewage concentrates was estimated by real-time RT-qPCR using a qScript XLT qPCR Toughmix system (Quantabio) in a Rotor-Gene Q instrument (Qiagen) following manufacturer’s instructions. Two samples from each England, Scotland and Pakistan and the sample from Senegal were analysed in duplicate using a two-step protocol including a reverse-transcription (RT) step and a complementary DNA (cDNA)-based qPCR step. Pan-EV primers targeting a conserved region of the 5’ untranslated region (forward primer, 5’-CCCTGAATGCGGCTAAT-3’; reverse primer, 5’-TGTCACCATAAGCAGCCA-3’; probe, 5’-[FAM]-ACGGACACCCAAAGTAGTCGGTTC-[TAM]-3’) [5] were used. A SuperScript III First-Strand Synthesis System (Invitrogen) and random hexamer primers were used for the RT step following manufacturer’s instructions, 10µl of purified RNA from each sewage concentrate were used per reaction. Following the RT step, 2-10 µl of random cDNA were used for the qPCR reaction with the following amplification conditions: 40 cycles of 95°C for 15 s, 50°C for 45 s, 61°C for 20 s and 72°C for 5 s. A standard curve for enterovirus RNA quantification was generated using serial dilutions of Coxsackievirus B5 purified RNA control (Vircell) containing 16,000 genome copies/µl. The results were expressed in Log_10_ EV genome copies/L of raw sewage.

**FIGURES**

**Supplementary Figure 1. Proportion of filtered NGS reads mapping to EV sequences from different strains present in control samples.**  Filtered reads from NGS analyses of Pan-EV RT-PCR products amplified from viral RNA purified from laboratory virus control mixtures were mapped to consensus sequences of each of the EV strains identified in the sample by *de novo* assembly. Results for different control mixtures (A to D) are reported. No EV strains were identified in any of the negative controls analysed.

**Supplementary Figure 2. Enterovirus RNA concentrations in sewage concentrates.** The concentration of EV RNA in sewage concentrates was estimated by real-time RT-qPCR. The results were expressed in Log10 EV genome copies/L of raw sewage. Values for each of the two determinations (Det 1 and Det 2) and the mean are shown.

**TABLES**

**Supplementary Table 1.** Details of sewage samples used in this study.

**Supplementary Table 2.** Genetic identity of EV strains identified in control samples.

**Supplementary Table 3.** EV serotype composition in sewage samples from different locations.

**REFERENCES**

1. Arita M, Kilpatrick DR, Nakamura T, et al. Development of an efficient entire-capsid-coding-region amplification method for direct detection of poliovirus from stool extracts. J Clin Microbiol **2015**; 53:73-8.

2. Majumdar M, Klapsa D, Wilton T, et al. Isolation of vaccine-like poliovirus strains in sewage samples from the UK. J Infect Dis **2017**.

3. Wheeler DL, Church DM, Lash AE, et al. Database resources of the National Center for Biotechnology Information: 2002 update. Nucleic Acids Res **2002**; 30:13-6.

4. Kroneman A, Vennema H, Deforche K, et al. An automated genotyping tool for enteroviruses and noroviruses. J Clin Virol **2011**; 51:121-5.

5. Brinkman NE, Fout GS, Keely SP. Retrospective Surveillance of Wastewater To Examine Seasonal Dynamics of Enterovirus Infections. mSphere **2017**; 2.
